# Effects of vildagliptin on wound healing and markers of inflammation in patients with type 2 diabetic foot ulcer: a prospective, randomized, double-blind, placebo-controlled, single-center study

**DOI:** 10.1186/s13098-022-00938-2

**Published:** 2022-12-02

**Authors:** Venkat N. Vangaveti, Shaurya Jhamb, Oliver Hayes, Julie Goodall, Jacqueline Bulbrook, Kelvin Robertson, Erik Biros, Kunwarjit S. Sangla, Usman H. Malabu

**Affiliations:** 1 Translational Research in Endocrinology and Diabetes, College of Medicine and Dentistry, James Cook University, 100 Angus Smith Drive, Douglas, QLD 4814 Australia; 2 Department of Endocrinology and Diabetes, Townsville University Hospital, Douglas, Townsville, QLD 4814 Australia; 3 Department of Pharmacy, Townsville University Hospital, Douglas, Townsville, QLD 4814 Australia

## Abstract

**Introduction:**

Diabetic foot ulcers (DFU) are one of the leading long-term complications experienced by patients with diabetes. Dipeptidyl Peptidase 4 inhibitors (DPP4is) are a class of antihyperglycemic medications prescribed to patients with diabetes to manage glycaemic control. DPP4is may also have a beneficial effect on DFU healing. This study aimed to determine vildagliptin's effect on inflammatory markers and wound healing.

**Trial design:**

Prospective, randomized, double-blind, placebo-controlled, single-center study.

**Methods:**

Equal number of participants were randomized into the treatment and placebo groups. The treatment was for 12 weeks, during which the participants had regular visits to the podiatrist, who monitored their DFU sizes using 3D camera, and blood samples were taken at baseline, six weeks, and 12 weeks during the study for measurement of inflammatory markers. In addition, demographic characteristics, co-morbidities, DFU risk factors, and DFU wound parameters were recorded.

**Results:**

50 participants were recruited for the study, with 25 assigned to placebo and 25 to treatment group. Vildagliptin treatment resulted in a statistically significant reduction of HBA1c (p < 0.02) and hematocrit (p < 0.04), total cholesterol (p < 0.02), LDL cholesterol (p < 0.04), and total/HDL cholesterol ratio (P < 0.03) compared to the placebo group. Also, vildagliptin had a protective effect on DFU wound healing, evidenced by the odds ratio (OR) favoring the intervention of 11.2 (95% CI 1.1–113.5; p < 0.04) and the average treatment effect on the treated (ATET) for vildagliptin treatment group showed increased healing by 35% (95%CI; 10–60, p = 0.01) compared to placebo with the model adjusted for microvascular complications, smoking, amputation, dyslipidemia, peripheral vascular disease (PVD) and duration of diabetes.

**Conclusions:**

Vildagliptin treatment was effective in healing DFU in addition to controlling the diabetes. Our findings support the use of DPP4is as a preferred option for treating ulcers in patients with diabetes. Further studies on a larger population are warranted to confirm our findings and understand how DPP4is could affect inflammation and DFU healing.

## Introduction

Diabetic patients are at exceptionally high risk for poor wound healing in general and foot ulcers in particular. The lifetime risk for developing chronic foot ulcers has been estimated to reach about 15–20% [1, 2]. Despite considerable advances in diabetic care, foot ulcers are responsible for a high number of lower limb amputations associated with decreased quality of life and increased mortality [2–4]. The significant risk factors for diabetic foot ulcers (DFU) are neuropathy and peripheral vascular disease. Diabetes induces complex vascular changes, promoting accelerated atherosclerosis and hypercoagulability, complicating foot ulcers, which can be assessed indirectly by many inflammatory markers [5].

Dipeptidyl peptidase-4 inhibitors (DPP4is) are a group of antihyperglycemic medications for managing type 2 diabetes mellitus. Several animal studies have suggested numerous beneficial anti-inflammatory effects of DPP4is beyond the effects on blood glucose alone [6–8]. Investigation into the anti-inflammatory property of DPP4i-vildagliptin in a human setting has shown benefits such as reducing oxidative stress and inflammation [9]. Treatment with a DPP4i may offer an attractive blood glucose reduction with the synergistic mechanism of action while exerting additional wound healing properties. Reduction of levels of inflammatory markers is of great clinical importance and has been shown to correlate with improvement of diabetic wound healing [10, 11].

In the present study, we focused on the potential anti-inflammatory properties of vildagliptin subjects with DFU. The critical representative serum markers for the study were chosen to represent alterations in inflammation markers, including interleukin 6 (IL-6), an inflammatory marker that markedly increased in individuals with DFU [10–12]. High circulating levels of high-sensitivity C-reactive protein (hs-CRP) and low levels of adiponectin, a hormone secreted from the adipose tissue with regulatory metabolic function, have also been reported to be associated with DFU. Reduction in hs-CRP levels represents a molecular hallmark of wound healing, while elevated adiponectin levels seem to protect against DFU progression [11]. Tumour necrosis factor-α (TNF-α) and transforming growth factor-beta 1 (TGF-β1) represent pro-inflammatory cytokines that are markedly increased and may serve as a good prognosis for diabetic foot syndrome [11, 13].

The primary objective of this study was to investigate whether Vildagliptin (100 mg/day) + standard of care (SOC) for DFU aids DFU healing compared to placebo + SOC over 12 weeks of treatment. In addition, we examined the effects of vildagliptin (100 mg/day) on circulating levels of interleukin-6 (IL-6), C-reactive protein (CPR), TNF-α, interleukin 1 beta (IL-1 beta), TGF-β, platelet reactivity, and adiponectin representing critical molecular markers of inflammation, thrombogenicity and wound healing, respectively. This study expanded on the known anti-glycemic effects of DPP4 inhibitors such as vildagliptin and informed on its beneficial effect on DFU wound healing, possibly by reducing inflammation, though the exact molecular mechanism remains to be identified.

## Study design

This study was a 12-week single-center, two-campus, randomized, double-blind, placebo-controlled clinical trial to compare Vildagliptin (100 mg/day) + SOC (intervention arm) with placebo + SOC (control arm), a ratio of 1:1. Informed consent was obtained from all participants prior to commencement of the study.

Study participants were randomised by an independent pharmacist using www.randomizer.org [14]. The pharmacist provided the vildagliptin and placebo tablets in a sealed envelope to the study nurse. The study nurse then provided tablets based on the randomization allocation sequentially to the participants.

## Inclusion criteria

Subjects ≥ 18 years of age diagnosed with type 2 diabetes on diet only or on any non-DPP4i medication were assessed. Patients with existing diabetes index foot ulcer grade A1 or higher, according to the University of Texas Wound Classification System of Diabetic Foot Ulcers, were included. A foot ulcer is defined as any full-thickness skin defect existing for a minimum of 14 days. In patients with multiple diabetic foot ulcers, the index foot ulcer is defined as the foot ulcer with the largest wound area at the time of inclusion. A suboptimal HBA1c ≥ 7.0% being an indication for use of vildagliptin was documented within 12 weeks prior to study inclusion or on the day of study inclusion.

## Exclusion criteria

The primary exclusion criteria included type 1 diabetes and current index foot ulcer of any non-diabetic pathophysiology. Also, major surgery up to 6 months before the day of enrolment or any planned surgery prior to study completion, including any major surgical intervention for the diabetic foot ulcer, were considered exclusion criteria. Patients with hypersensitivity to vildagliptin, one of its excipients, or any other contraindication for vildagliptin use, including a pre-treatment ALT or AST > 3 × ULN (upper limit of normal), were excluded. Patients undergoing treatment with normothermic or hyperbaric oxygen therapy, enzymatic debridement, having Charcot's foot, renal impairment (defined as eGFR less than 60 ml/min/1.73 m^2^ for more than three months), and on GLP-1 analogs or DPP4is and on any other clinical trials were also excluded.

All participants were to remain on study medication and followed up for 12 weeks even if their index ulcer had completely healed prior to the end of the 12-week observation period.

This study was performed using the national and international guidelines: the ICH/GCP guidelines (Guidance on Good Clinical Practice [CPMP/GCP/135/95] and Guidance on Good Clinical Practice [CPMP/GCP/135/95] annotated with Therapeutic Goods Administration (TGA) comments [DSEB, July 2000]), the NHMRC National Statement on Ethical Conduct in Human Research (2007) and all other applicable Australian Commonwealth, State or Territory laws or guidelines of Regulatory Authorities as well as following the ethical principles that have their origin in the Declaration of Helsinki. The final study protocol and the final version of the written informed consent form, case report form (CRF), and patients' home blood glucose monitoring diaries were approved in writing by an independent ethics committee (IEC) HREC/13/QTHS/65 and the Trial Registration: ACTRN12613000418774. Written informed consent was obtained from all subjects prior to commencement of the study.

## Laboratory procedures

Morning fasting venous blood samples were taken for determining levels of inflammatory markers and was centrifuged after 30 min of collection for 12 min at 3000 rpm. 0.1 ml of venous plasma was stored at − 80 °C for analysis as a batch to minimize variance. Serum levels of various candidates were measured using the ELISA kit as per manufacturer’s instructions. Details of ELISA kits were as follows: Interleukin 6, Interleukin 1β, Tumor Necrosis Factor-α, adiponectin, C-reactive protein and Transforming growth factor β (Abcam, Australia).

## Sample size calculations and data analysis

The sample size calculation in this study was conducted to demonstrate superior efficacy of vildagliptin (100 mg/day) + SOC over placebo + SOC on wound healing from randomization to week 12. The appropriate sample size was attained by assuming an anticipated difference in wound healing of 20% for diabetic foot ulcer healing at week 12 between the intervention and control groups. A power set to 80% yielded a calculated sample size of 22 wounds per arm. Adding an estimated attrition rate of close to 15% = 3 ulcers per arm led to an overall planned sample size of a minimum of 25 wounds per arm and 50 wounds in total.

## Data analysis

For patients with post-randomization data, the last study assessment was carried forward as their final assessment for analyses. These served as conservative estimates since the patients were expected to improve over time. Descriptive statistics were provided for all measured indices and clinical/demographic characteristics. All data were analyzed using SPSS Version 25 or Stata 16. Tests for normality were performed, and based on the outcome, parametric or nonparametric tests were used to determine the differences between the groups. The results were reported as mean ± standard deviation or median and interquartile range where appropriate. ANCOVA was used to determine the effect of treatment on variables at 12 weeks. Chi-squared analysis was performed for categorical variables. Binary logistic regression analysis was used to determine the factors for wound healing with confounding factors of microvascular complications, smoking, amputation, dyslipidemia, PVD, and year of diabetes included in the model. The average treatment effect (ATE) was calculated using a logit model with the inverse probability weights regression adjustment. P-value < 0.05 was to be considered significant.

## Results

**Fig. 1:**
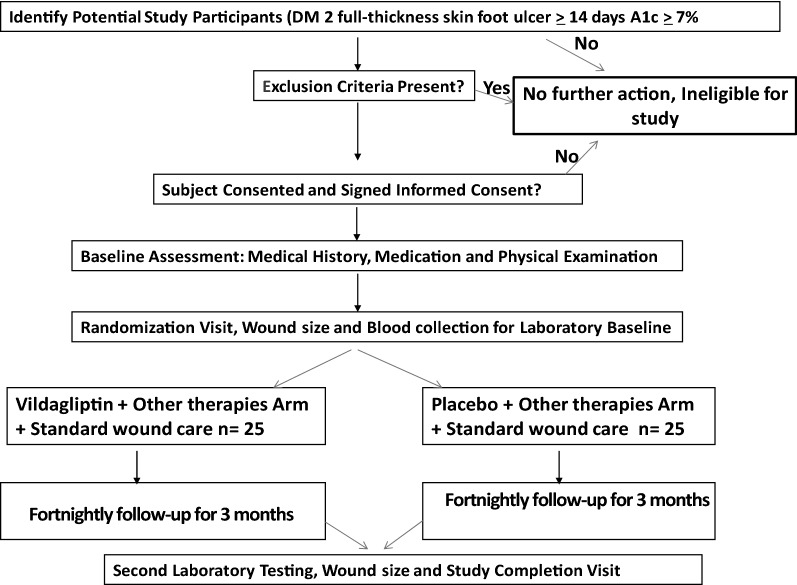
Study design

**Fig. 2:**
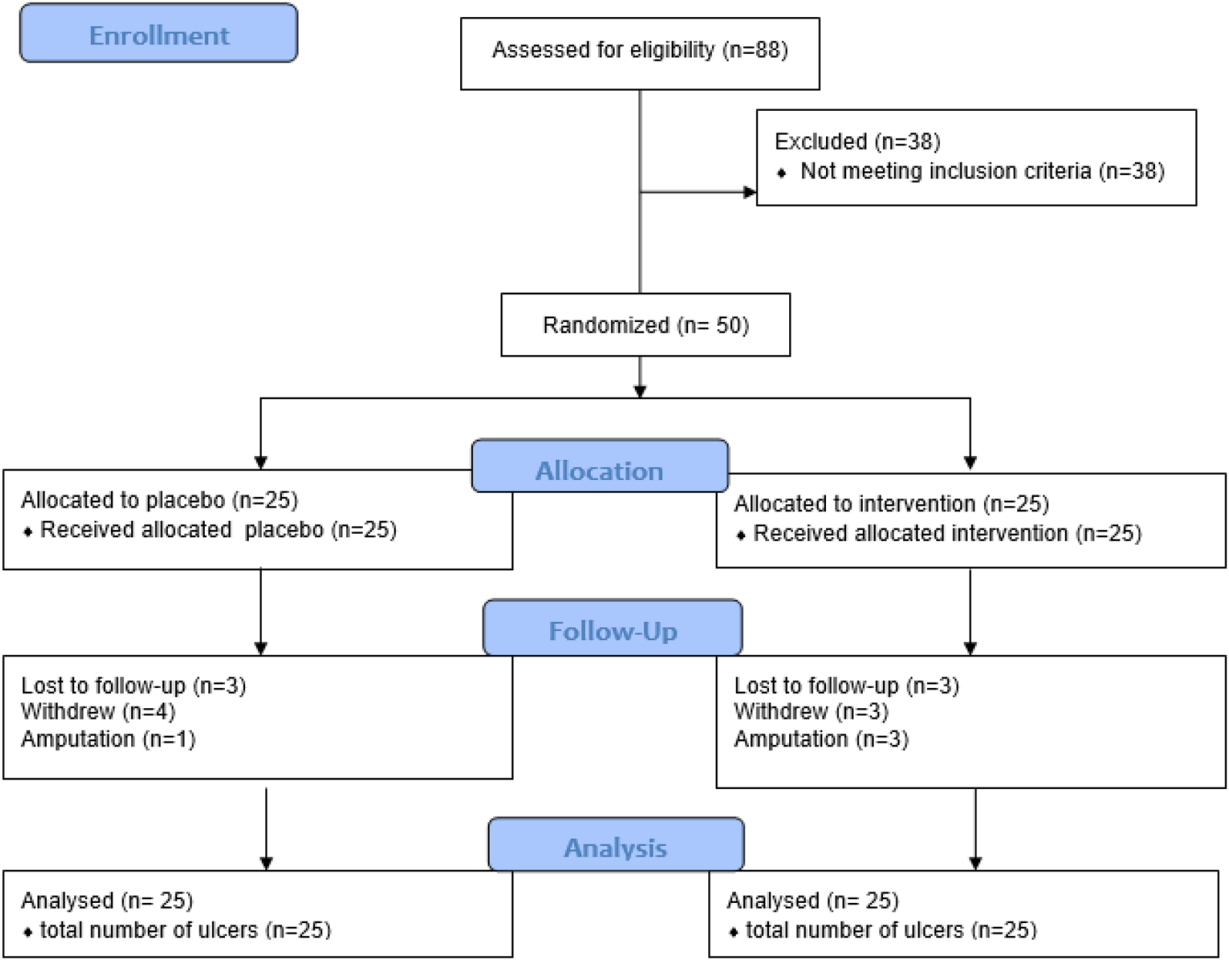
Study analysis: CONSORT flow diagram

Eighty-eight patients were screened for eligibility, of which 38 did not meet the inclusion criteria. Fifty patients with proven DFU were recruited and randomized [14] in a 1:1 ratio to either vildagliptin and other antidiabetic therapies (n = 25) or placebo and other antidiabetic therapies (n = 25) for 12 weeks, as shown in Figs. 1 and 2. Seven participants withdrew during the study, 6 were lost to follow-up, and 4 had an amputation. An intention-to-treat analysis was undertaken for 50 ulcers n = 25 and n = 25 of the randomized placebo and vildagliptin groups, respectively (Fig. 2). With some patients having multiple ulcers, each group's total number of ulcers was 25. The demographic characteristics such as age, gender distribution, BMI, and duration of diabetes were all similar in the randomized groups (Table 1).

**Table 1:** Baseline characteristics of study population

	Control group (n = 25)	Vildagliptin group (n = 25)	*P*
Mean age (years)	63.0 ± 10.0	64.0 ± 10.1	0.81
Sex (M/F)	23/2	18/7	0.20
BMI (kg/m^2^)	35 ± 8.3	36.4 ± 8.0	0.90
Diabetes duration (years)	16.6 ± 10.0	23.2 ± 7.0	0.20
Waist-to-hip ratio	0.91 ± 0.06	0.92 ± 0.09	0.74
Systolic blood pressure (mmHg)	137.0 ± 23	138.0 ± 17.0	0.81
Diastolic blood pressure (mmHg)	80.4 ± 9.3	71.3 ± 9.4	**0.003**
Heart rate (bpm)	83 ± 26	84 ± 24	0.92
Risk factors (%)			
Dyslipidemia (%)	16 (64)	19 (76)	0.41
Retinopathy (%)	12 (48)	12 (48)	1.00
Nephropathy (%)	8 (32)	6 (24)	0.71
CAD	5 (20)	6 (24)	1.00
CVA/tIA	4 (16)	3 (12)	1.00
PVD	8 (32)	7 (28)	1.00
PVD vascular surgery	2 (8)	2 (8)	1.00
Amputation (non-traumatic)	11 (44)	11 (44)	1.00
Hypertension	18 (72)	19 (76)	0.70
Neuropathy (%)	17 (68)	19 (76)	0.50
Alcohol (%)	7 (28)	12 (48)	0.14
Smokers (%)	6 (24)	6 (24)	1.00
Therapy			
Insulin (%)	17 (68)	17 (68)	1.00
OHGs	22 (88)	19 (76)	0.50
Metformin (%)	17 (65.4)	20 (62.5)	
Gliclazide (%)	3 (11.5)	8 (25.0)	
Dapagliflozin (%)	6 (23.1)	4 (12.5)	

Data are means ± SD or n (%). *BMI* body mass index, *CAD* coronary artery disease, *CVA/tIA* cerebrovascular accident/transient ischemic attack, *PVD* peripheral vascular disease, *OHG* oral hypoglycaemic drugs

The clinical, biochemical parameters and inflammatory markers (baseline, week 6, and week 12) were analyzed at the end of the study. The two groups had no significant differences in most metabolic risk factors. Both the groups had a similar distribution of the other insulin and oral hypoglycaemic medications. The baseline characteristics of the biochemical parameters, inflammatory markers, and ulcer dimensions in the study population were similar in both control and vildagliptin-treated groups (Table 1).

Subjects on vildagliptin had lower levels of A1c; p < 0.02, cholesterol; p < 0.02, total/HDL cholesterol ratio; P < 0.03 and LDL cholesterol; p < 0.04 compared with controls. Other inflammatory markers, including IL-6, showed no significant differences (Table 2). C-reactive protein (CRP), WBC, neutrophils, ulcer length and width, and surface area were also reduced in the vildagliptin group compared to placebo (p < 0.05) (Table 2).

**Table 2:** Differences in clinical parameters at week 12 with control vs vildagliptin, mean (SEM) reported

Characteristics	Control	Vildagliptin	Mean difference	P
Laboratory results				
HBA1c	9.1 ± 0.3	8.1 ± 0.3	1.0	**0.02**
Protein (total)	73.2 ± 1.1	71 ± 1.2	2.3	0.20
Albumin	37.0 ± 1.0	39.0 ± 1.1	− 2.1	0.20
Globulin	35.7 ± 1.0	33.4 ± 1.0	2.3	0.07
Urea/creat	87.0 ± 5.6	82.4 ± 6.1	4.6	0.60
eGFR	69.5 ± 2.3	71.4 ± 2.3	− 1.8	0.60
WBC	9.1 ± 0.4	8.0 ± 0.4	1.3	0.06
Neutrophils	6.1 ± 0.4	5.0 ± 0.4	1.2	0.06
Haematocrit	0.39 ± 0.07	0.37 ± 0.07	0.02	**0.04**
Hb	130.0 ± 3.0	123.0 ± 3.0	7.0	0.09
Platelets	268.0 ± 20.1	265.0 ± 19.2	3.0	1.00
Cholesterol (mmol/L)	4.3 ± 0.3	3.4 ± 0.24	0.9	**0.02**
Triglycerides (mmol/L)	2.5 ± 0.4	2.1 ± 0.3	0.4	0.40
HDL (mmol/L)	1.0 ± 0.04	1.0 ± 0.03	0.005	1.00
Total/HDL ratio	5.0 ± 0.3	4.0 ± 0.3	1.0	**0.03**
LDL Chol (mmol/L)	2.3 ± 0.24	1.5 ± 0.23	0.8	**0.04**
VLDL	1.0 ± 0.1	0.8 ± 0.1	0.2	0.13
Urine creatinine (mg/dL)	11.9 ± 1.6	7.7 ± 1.4	4.2	0.08
Free T4	12.0 ± 0.5	11.5 ± 0.4	0.50	0.50
FreeT3	4.5 ± 0.2	4.5 ± 0.20	− 0.03	1.00
TSH	1.6 ± 0.3	1.7 ± 0.30	− 0.05	1.00
Inflammatory markers				
IL-6 (pg/ml)	7.1 ± 2.5	10.0 ± 3.0	− 2.9	0.50
IL-1β (pg/ml)	150.0 ± 5.6	136.0 ± 5.6	12.8	0.12
TNF-α (pg/ml)	30.0 ± 1.0	29.0 ± 1.0	0.20	1.00
Adiponectin (ng/ml)	11,822.0 ± 2584.0	13,138.0 ± 2671.0	− 1316.0	1.00
CRP (mg/ml)	0.12 ± 0.02	0.10 ± 0.02	0.05	0.06
TGF-β (pg/ml)	1053.0 ± 196.0	952.0 ± 202.0	101.2	0.70
Ulcer parameters				
Ulcer length mm	12.2 ± 2.3	8.1 ± 2.3	4.0	0.30
Ulcer width mm	12.4 ± 2.3	8.1 ± 2.3	4.3	0.20
Ulcer surface area mm^2^	270.0 ± 94.0	128.1 ± 94.0	141.0	0.30
Ulcer depth mm	2.1 ± 0.5	1.4 ± 0.5	0.7	0.30

*WBC* white blood cells, *HDL* high density lipoprotein, *LDL* low density lipoprotein, *VLDL* very low density lipoprotein, *TSH* thyroid stimulating hormone, *IL-6* interleukin

At the end of 12-week treatment period, of the total of 50 ulcers, 14 (28%) were completely healed. The factors for wound healing were determined using multivariate logistic regression analysis. The analysis revealed that vildagliptin has a favorable OR of 11.2 (95% CI 1.1–113.5, p < 0.04) with the model adjusted for microvascular complications, smoking, amputation, dyslipidemia, PVD, and duration of diabetes (Table 3). In addition, the average treatment effect on the treated (ATET) for the vildagliptin treatment group increased healing by 35% (95%CI; 10–60), p = 0.01 compared to placebo; adjusted for microvascular complications, smoking amputation, dyslipidemia, PVD, and year of diabetes.

**Table 3:** Multivariate logistic regression revealed no significant variable directly affecting wound healing

Characteristics	Ulcer unhealed n = 36	Ulcer healed n = 14	OR 95%CI Unadjusted P value	OR 95%CI Adjusted P value
Placebo	20 (55.5)	5 (35.7)		
Vildagliptin	16 (44.4)	9 (64.3)	2.2 (0.6–8.0); 0.21	11.2 (1.1–113.5); **0.04**
Microvascular complication*	30 (83)	10 (71)	0.5 (0.11–2.2); 0.43	0.9 (0.03–24.0); 0.90
Smoking	10(28)	2(14)	0.4 (0.08–2.3); 0.30	0.5 (0.03–8.8); 0.50
Amputation	16 (44)	6 (42)	1.0 (0.3–3.2); 1.00	0.7 (0.04–11.5); 0.20
Dyslipidaemia	27 (75)	8 (57)	0.4 (0.1–1.6); 0.20	0.2 (0.02–2.3); 0.20
Peripheral vascular disease	10 (28)	5 (35)	0.7 (0.2–2.6); 0.60	0.3 (0.3–3.3); 0.33
Years of diabetes	19.3 ± 10.0	21.2 ± 7.5	1.0 (0.9–1.1) 0.60	0.9 (0.8–1.1); 0.70

*Microvascular complication included retinopathy, nephropathy, neuropathy

## Discussion

We have shown that vildagliptin treatment over 12 weeks can aid wound healing. This finding is in line with Marfella et al.*’s* previous report, that DDP4is may facilitate healing of chronic foot ulcers [15]. Our analysis showed the beneficial effect of vildagliptin in healing ulcers over 12 weeks with a favorable odds ratio of more than 11. Although DPP4is have shown promising wound healing properties [16], this study examined the effect of vildagliptin treatment over 12 weeks in patients with type 2 diabetes undergoing their SOC for DFU, representing a relatively short intervention period. Furthermore, vildagliptin treatment is efficacious in reducing HBA1c levels, whether treated as monotherapy [17] or combination therapies [18]. This study confirmed other metabolic and systemic effects of vildagliptin, such as improving lipids profile [19] and reducing systemic inflammation, thus aligned with previous findings [20–22]. Furthermore, our data substantiate previous findings since the study was a randomized clinical trial and double-blinded with one team involved in running the trial thus eliminating the possible inter-investigator variation.

Moreover, we attempted to quantify vildagliptin's potential effect on DFU healing and estimated a 35% increase in healing capacity compared to controls. This finding represents the clinically meaningful properties of vildagliptin in managing DFU [23]; however, the use of DPP4is for DFU treatment might not be without complications [24–26]. For instance, the U.S. Food and Drug Administration (FDA) warns that these drugs may cause joint pain that can be severe and disabling [27], suggesting that possible side effects should be borne in mind when using DPP-4is. However, we did not record any severe side effects associated with vildagliptin, possibly acknowledging only short-term use of this drug.

In conclusion, the vildagliptin treatment in DFU patients improves wound healing with an associated reduction in some inflammatory biomarkers. Our findings support that DPP4is inhibitors could be a preferred option for treating ulcers in patients with diabetes; however, long-term effects need to be determined precisely, including an extended follow-up to determine DFU reoccurrence following the vildagliptin treatment.

## Data Availability

All data generated or analysed during this study are included in this published article.
